# Impact of Lanthanide (Nd^3+^, Gd^3+^, and Yb^3+^) Ionic Field Strength on the Structure and Thermal Expansion of Phosphate Glasses

**DOI:** 10.1021/acs.jpcb.3c07767

**Published:** 2024-03-18

**Authors:** José A. Jiménez, Richard Amesimenu, Madison Thomas

**Affiliations:** Department of Biochemistry, Chemistry, and Physics, Georgia Southern University, Statesboro, Georgia 30460, United States

## Abstract

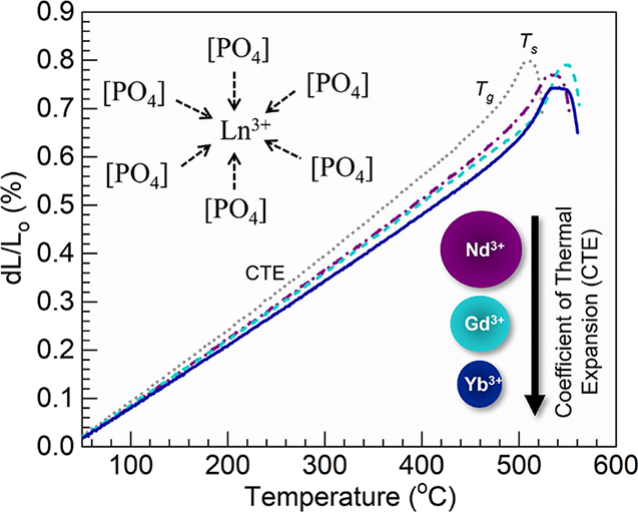

Phosphate glasses containing Nd^3+^, Gd^3+^, and Yb^3+^ as lanthanide ions are attractive for applications in laser materials, phototherapy lamps, and solar spectral converters. The composition–structure–property relation in this type of glass system is thus of interest from fundamental and applied perspectives. In this work, the impact of the differing ionic radius of Nd^3+^, Gd^3+^, and Yb^3+^ and consequent field strength on the physical properties of phosphate glasses is investigated, focusing ultimately on thermal expansion effects. The glasses were made by melting with a fixed concentration of the lanthanide ions having 50P_2_O_5_–46BaO–4Ln_2_O_3_ nominal compositions (mol %) with Ln = Nd, Gd, and Yb. The investigation encompassed measurements by X-ray diffraction (XRD), optical spectroscopy, density, X-ray photoelectron spectroscopy (XPS), Raman spectroscopy, and dilatometry. XRD supported the amorphous nature of the glasses, whereas absorption and photoluminescence spectra showed the optical features of the Nd^3+^, Gd^3+^, and Yb^3+^ ions in the glasses. Oxygen speciation by XPS indicated an increase in nonbridging oxygens for the larger radii Nd^3+^ and Gd^3+^ ions relative to the host, contrasting with Yb^3+^. Phosphorus XPS analysis further supported the hypothesis that the P 2p binding energies of the glasses increased with the cation field strength of the lanthanides. The Raman spectra were interpreted based on glass depolymerization effects and the impact of Ln^3+^ ions with high field strength. Particularly, the band position of the symmetric out-of-chain nonbridging oxygen stretch, ν_s_(PO_2_^–^), shifted to higher frequencies correlating with the Ln^3+^ field strength. Dilatometry ultimately revealed a steady decrease in the coefficient of thermal expansion for the glasses, which correlated linearly with Ln^3+^ field strengths and thus indicated to sustain increased glass rigidities. The various analyses performed thus illuminated the structural foundation of the thermomechanical behavior of the glasses connected with changes in the Ln^3+^ field strengths.

## Introduction

Glasses activated with trivalent lanthanide ions (Ln^3+^) have been the subject of extensive research given the diversity of radiative electronic transitions making them valuable for a variety of applications as optical materials.^1−5^ Among the different trivalent lanthanides, Nd^3+^,^6−8^ Gd^3+^,^9,10^ and Yb^3+^,^11−13^ are attractive for use in laser materials, phototherapy lamps, and solar spectral converters. With respect to these, a distinctiveness in terms of their relative positions in the periodic table of elements is that the three lanthanides are far apart with atomic numbers of 60 (Nd), 64 (Gd), and 70 (Yb). Hence, the size of the technologically relevant Nd^3+^ ([Xe] 4f^3^), Gd^3+^ ([Xe] 4f^7^), and Yb^3+^ ([Xe] 4f^3^) ions varies significantly in accord with the lanthanide contraction.^14,15^ This in turn translates as increased ionic field strengths (*F*) commonly expressed in Coulomb’s law framework as
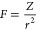
1where *Z* is the cation charge and *r* the ionic radius.^15,16^ Variation in ionic radii of lanthanides and consequently the field strengths have thus been considered to be capable of impacting various properties of the glass host. In this context, Sendova et al.^16^ studied Ln^3+^-doped phosphate glasses by differential scanning calorimetry and reported a correlation of the glass transition activation energy with the trivalent lanthanide radius. Hayden et al.,^17^ Campbell,^18^ and Campbell and Suratwala^19^ reported extensively the effect of various alkali and alkaline earth cations in neodymium laser glasses and reported correlations with the average field strength of the cations for properties such as thermal expansion, Young’s modulus, refractive index, and emission cross sections. The dependence of thermal and mechanical properties with cation field strength has been also investigated in other glass systems such as Ln-containing Si–Mg–O–N and Si–Al–O–N glasses by Lofaj et al.^15^ and Menke et al.,^20^ respectively. However, the connection with fundamental structural properties assessed, for instance, through spectroscopic techniques is still lacking. Further research is then desired to assess comprehensively the structure–property relationship in glasses containing different technologically relevant Ln^3+^ ions with varying radii/field strengths. Of great value in this sense is wide-ranging spectroscopic characterizations, which help elucidate the different contributions of the various constituents to glass structure and the consequent impact on thermomechanical properties of interest.

The present work was then undertaken with the purpose of evaluating the composition–structure relationship in Ln^3+^-containing phosphate glasses, focusing on thermal/dilatometric properties scarcely scrutinized. Using as a starting point the high metal solubility 50P_2_O_5_–50BaO glass matrix previously considered in the context of various photonic applications,^1,8,10,13^ the glasses were made with 50P_2_O_5_–46BaO–4Ln_2_O_3_ (mol %) nominal compositions where Ln = Nd, Gd, and Yb. The glasses were synthesized by the melt-quenching technique, followed by an experimental investigation encompassing measurements by X-ray diffraction (XRD), optical absorption, photoluminescence (PL) spectroscopy, density, X-ray photoelectron spectroscopy (XPS), Raman spectroscopy, and dilatometry. The various parameters extracted from measurements were consequently examined in the context of the glass structure and differing cation field strengths seeking insights into the physical origin of the thermomechanical properties of the Ln^3+^-activated glasses.

## Experimental Section

### Glass Synthesis

The glasses were prepared by the melt-quench technique with 50P_2_O_5_–46BaO–4Ln_2_O_3_ nominal compositions (mol %) with Ln = Nd, Gd, and Yb. The concentration of the lanthanide oxides was chosen such that it was significant enough to produce considerable changes in the properties studied while avoiding the risk of crystallization during quenching. The undoped 50P_2_O_5_–50BaO glass was also prepared as a reference. Reagents used as raw materials were high-purity P_2_O_5_ (≥98%), BaCO_3_ (99.8%), Nd_2_O_3_ (99.99%), Gd_2_O_3_ (99.9%), and Yb_2_O_3_ (99.99%). Batch materials were weighed in the appropriate quantities (about 25 g batches), thoroughly mixed, and melted under an ambient atmosphere in porcelain crucibles at 1150 °C within 15–20 min. The melts were swirled to ensure homogeneity and quenched by being poured onto heated steel molds. The glasses were annealed below the glass transition temperature at 420 °C for 3 h to remove mechanical/thermal stress. The glasses were cut and polished to about 1 mm thick slabs for spectroscopic measurements. Glass samples were also quenched in cylindrical shapes and cut to a length (*L*) of about 2.54 cm for dilatometric measurements. The glass codes and respective nominal compositions are summarized in Table 1.

**Table 1:** Glass Codes and Nominal Compositions of the Glasses Synthesized

glass	P_2_O_5_ (mol %)	BaO (mol %)	Nd_2_O_3_ (mol %)	Gd_2_O_3_ (mol %)	Yb_2_O_3_ (mol %)
Ba	50	50			
Nd	50	46	4		
Gd	50	46		4	
Yb	50	46			4

### Measurements

Powder XRD was performed to verify the amorphous nature of the glasses (crushed to powder by a mortar and pestle) with a PANalytical Empyrean X-ray diffractometer operating at room temperature (RT) using the Mo-*K*_α_ radiation (λ = 0.71 Å) available. The acceleration voltage and current used were 60 kV and 40 mA, respectively.

Optical absorption measurements were performed at RT on the ∼1 mm thick glass samples fixed on a sample holder with an Agilent UV–vis–NIR Cary 5000 double-beam spectrophotometer; the reference in the measurements was air.

PL spectra were collected at RT under steady-state conditions with a Horiba Fluorolog-QM spectrofluorometer equipped with a continuous illumination Xe lamp and an InGaAs detector.

The densities were determined for the various glasses by the Archimedes principle with a Mettler-Toledo XSR analytical balance using distilled water as the immersion liquid. Measurements were done in triplicate, and the averages were reported (uncertainties in third decimal place). Other physical parameters judged useful for characterizing the glasses were also calculated in agreement with corresponding formulas.^21,22^ The average molar mass (*M*_av_) was calculated by
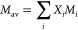
2where *X*_*i*_ and *M*_*i*_ are the mole fraction and molar mass of the *i*th component, respectively. From the measured densities (ρ), the molar volumes (*V*_m_) were obtained as
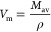
3The concentration (*N*) of Ln^3+^ ions in the glasses was calculated with the corresponding mole fractions (*X*), the glass densities (ρ), and the average molar masses (*M*_av_) according to
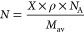
4where *N*_A_ is Avogadro’s constant. The mean interionic distances (*d*) between Ln^3+^ ions were then calculated from the following relation
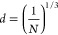
5

XPS measurements were carried out on the polished glass slabs at RT using a Thermo K Alpha XPS system with a monochromatic Al-*K*_α_ X-ray source (1486 eV). A Flood gun (low energy ionized argon beam) was used to neutralize charging effects since samples are nonconductive. All samples were sputter cleaned with an argon ion beam for 30s to remove surface contaminants before collecting data. The adventitious carbon (C 1s) peak at 284.8 eV was used as an internal reference for peak position determinations. The binding energies for individual O 1s and P 2p contributions were determined by fitting procedures using Gaussian–Lorentzian peak shapes.

Raman spectra were recorded at RT using polished glass slabs with a Thermo Scientific DXR Raman microscope operating at 532 nm and a power of 10 mW. A 10× MPlan objective was employed for data collection with the acquisition time for each spectrum set at 100 s. Baseline subtraction was performed using OriginPro, after which the spectra were normalized for comparison.

Dilatometry measurements were carried out on the 2.54 cm-long glass cylinders in an Orton dilatometer (Model 1410B) operating at a heating rate of 3 °C/min under an ambient atmosphere.

## Results and Discussion

### XRD and Optical Characterization

**Figure 1:**
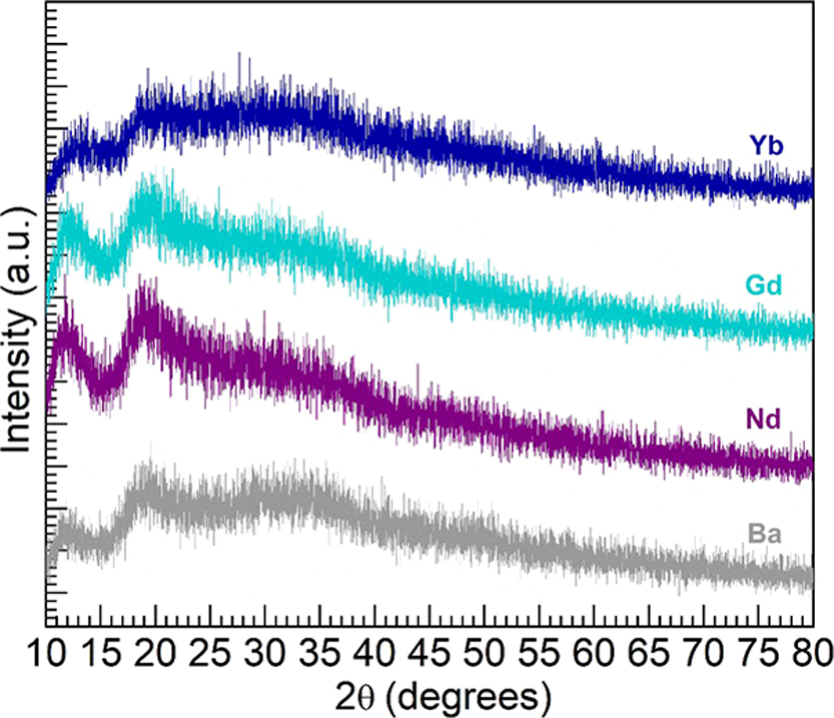
XRD patterns obtained with Mo-*K*_α_ radiation within the 10° ≤ 2θ ≤ 80° range for the various glasses synthesized.

Starting in this section, we consider characterization results concerning the amorphous structure of the glasses and the optical properties assessed by absorption and PL spectroscopy. Figure 1 shows the XRD patterns obtained for the different glasses with Mo-*K*_α_ radiation recorded in the 10° ≤ 2θ ≤ 80° range. The glasses exhibit qualitatively similar humps due to diffuse scattering stemming from long-range structural disorder. These appear however shifted toward lower angles compared to the typically obtained using Cu-*K*_α_ radiation (1.54 Å)^22,23^ in connection with the shorter wavelength of Mo-*K*_α_ X-ray photons (0.71 Å). The diffractograms in Figure 1 still do not show distinct crystallization peaks. Thus, the noncrystalline nature of the 50P_2_O_5_–50BaO and 50P_2_O_5_–46BaO–4Ln_2_O_3_ (Ln = Nd, Gd, Yb) glasses synthesized is supported.

**Figure 2:**
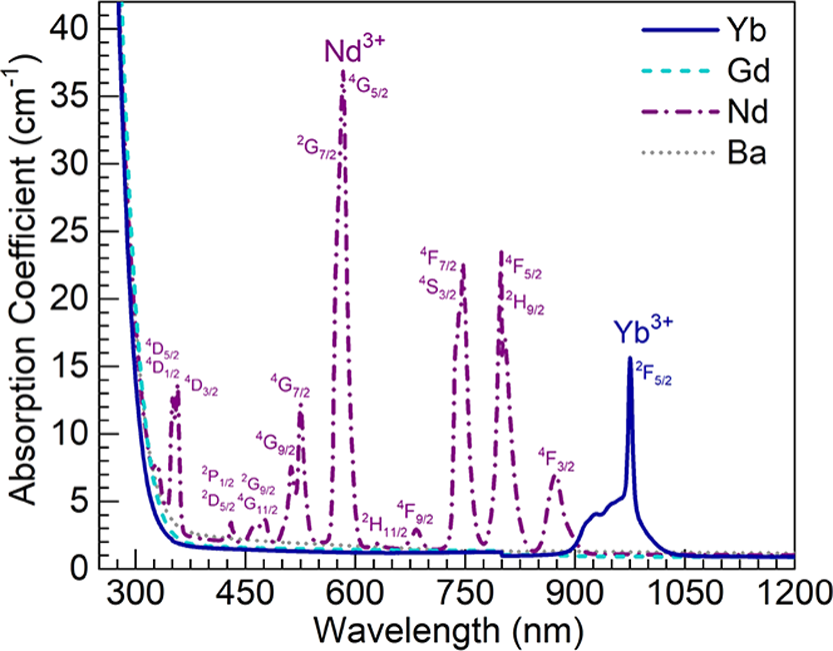
UV–vis–NIR absorption spectra for the different glasses; some excited states associated with prominent absorption in Nd^3+^ and Yb^3+^ ions from the ^4^I_9/2_ and ^2^F_7/2_ ground states, respectively, are indicated.

The optical absorption spectra obtained for the four glasses under consideration are shown in Figure 2. The Yb glass displays conspicuously the ^2^F_7/2_ → ^2^F_5/2_ near-infrared (NIR) absorption peak of Yb^3+^ ions around 975 nm.^12,13^ The Nd glass exhibits the different transitions spanning from the NIR to the visible range, most prominently the absorption peak around 583 nm in connection with ^4^I_9/2_ → ^4^G_5/2_ + ^2^G_7/2_ transitions in Nd^3+^ ions.^3,7,8^ The Gd glass, on the other hand, shows an absorption profile in good resemblance with the undoped Ba glass host. This is because the Gd^3+^ electronic transitions responsible for light absorption lie in the UV within the region where the glass host also absorbs.^9,10^

**Figure 3:**
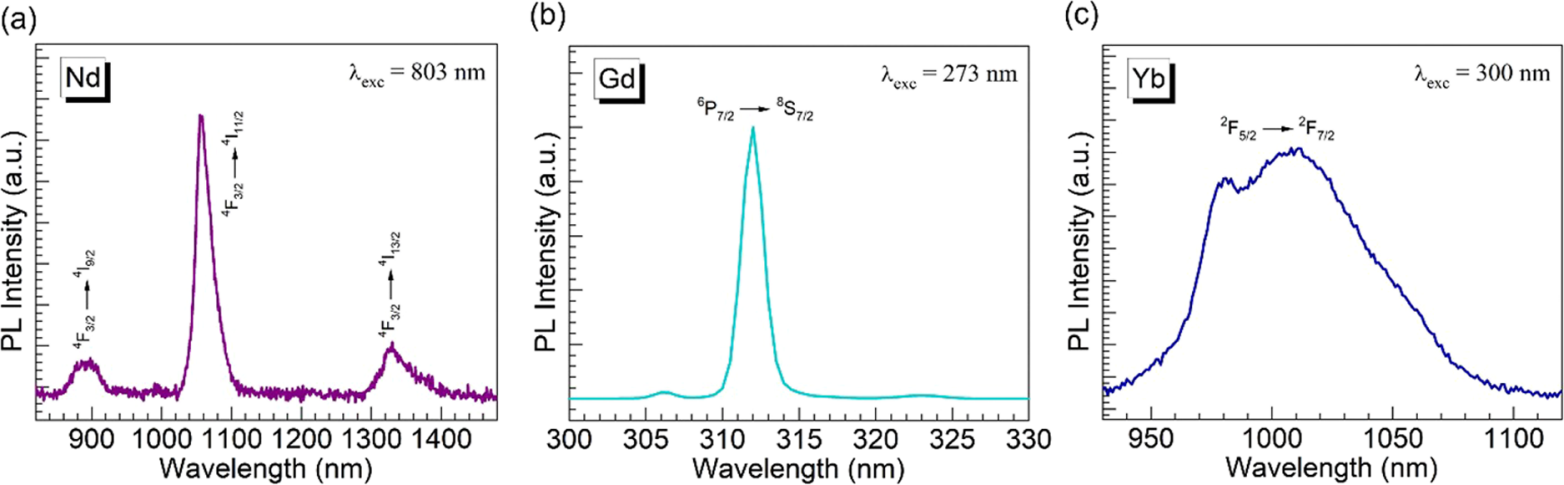
PL spectra recorded for the Ln^3+^-containing glasses: (a) Nd glass (λ_exc_ = 803 nm); (a) Gd glass (λ_exc_ = 273 nm); and (c) Yb glass (λ_exc_ = 300 nm).

To optically characterize the Ln^3+^-doped glasses more fully, PL spectra were obtained for the Nd, Gd, and Yb glasses under suitable excitation conditions. In Figure 3a, the PL emission spectrum of the Nd glass is presented, which was obtained under the excitation of ^4^I_9/2_ → ^4^F_5/2_ + ^2^H_9/2_ transitions in Nd^3+^ ions at 803 nm.^8^ The typical Nd^3+4^F_3/2_ → ^4^I_9/2_, ^4^I_11/2_, ^4^I_13/2_ NIR transitions are observed around 890, 1056, and 1330 nm, respectively.^7,8^ The Gd glass was excited at 273 nm to promote ^8^S_7/2_ → ^6^I_J_ transitions in Gd^3+^ ions, and the spectrum obtained is shown in Figure 3b. It shows the characteristic UV type B emission peak of Gd^3+^ ions around 312 nm corresponding to ^6^P_7/2_ → ^8^S_7/2_ transitions.^9,10^ Finally, Figure 3c shows the PL spectrum of the Yb glass recorded under the excitation of O^2–^–Yb^3+^ charge transfer transitions at 300 nm.^13^ It thus exhibits the broad NIR ^2^F_5/2_ → ^2^F_7/2_ emission from Yb^3+^ ions.^12,13^ Overall, the XRD and optical spectroscopy data support the effective synthesis of the glasses. We then proceeded to evaluate glass densities and basic physical properties next.

### Density and Basic Physical Properties

The densities and other physical parameters calculated for the four glasses under study are given in Table 2. Also presented in Table 2 are the ionic radii employed for the evaluation and the corresponding field strengths calculated by eq 1. The ionic radii are based on Shannon^14^ for a coordination number (CN) of 8 for Ba^2+^ in accord with Hoppe et al.,^24^ whereas a CN of 6 was assumed for the Ln^3+^ ions following the works of Karabulut et al.^25^ and Zou et al.^26^ The resulting field strengths obtained in this way for Ba^2+^, Nd^3+^, Gd^3+^, and Yb^3+^ were 0.992, 3.105, 3.410, and 3.982 Å^–2^, respectively. The values for Nd^3+^, Gd^3+^, and Yb^3+^ coincide with that reported by Lofaj et al.^15^ in their evaluation of rare-earth-doped oxynitride glasses which considered 6-fold coordination for the Ln^3+^ ions as well.

**Table 2:** Parameters Related to the Basic Physical Properties of the Different Glasses along with Cation Radii, *r*, Based on Shannon^14^ for Eightfold-Coordinated Ba^2+^ Ions^24^ and Sixfold-Coordinated Ln^3+^ Ions,^25,26^ and the Corresponding Calculated Field Strengths

parameter	glass			
	Ba	Nd	Gd	Yb
density, ρ (g/cm^3^)	3.700	3.780	3.826	3.794
average molar mass, *M*_av_ (g/mol)	147.64	154.96	156.00	157.27
molar volume, *V*_m_ (cm^3^/mol)	39.90	40.99	40.77	41.45
Ln^3+^ concentration, *N* (× 10^20^ ions/cm^3^)		11.75	11.82	11.62
Ln^3+^–Ln^3+^ mean distance, *d* (Å)		9.48	9.46	9.51
[O]/[P]	3.00	3.08	3.08	3.08
cation radius,^a^*r* (Å)	1.42	0.983	0.938	0.868
cation field strength, *F* = *Z*/*r*^2^ (Å^–2^)	0.992	3.105	3.410	3.982

a From Shannon.^14^

The densities are observed in Table 2 to increase with the 4 mol % Ln_2_O_3_ added at the expense of BaO for Ln = Nd and Gd, namely, from 3.700 g/cm^3^ for the Ba glass up to 3.826 g/cm^3^ for the Gd glass. The Ba glass density is close (although marginally higher) to the reported for an equivalent glass prepared using an alumina crucible of 3.680 g/cm^3^.^22^ The density of the Yb glass of 3.794 g/cm^3^, although higher than those of the Ba and Nd glasses, is nonetheless somewhat lower than the density obtained for the Gd glass. While the average molar masses increase steadily as anticipated, the molar volumes are seen in Table 2 to fluctuate, in accord with different densities. However, the Yb glass exhibits the highest value, 41.45 cm^3^/mol. The Ln^3+^ concentrations are similar for the Ln-containing glasses within the 11.62 × 10^20^–11.82 × 10^20^ ions/cm^3^ range, leading to the values for the mean Ln^3+^-Ln^3+^ interionic distances being around 9.5 Å. Comparable values were also found for a glass with 50P_2_O_5_–46BaO–4Eu_2_O_3_ nominal composition prepared using a high-purity alumina crucible.^22^ This provides confidence in the structural and thermal property evaluation as it suggests that the Ln^3+^ ions are similarly embedded in the glasses. The nominal [O]/[P] ratio in Table 2 changes from the metaphosphate in the 50P_2_O_5_–50BaO binary glass toward the polyphosphate type where [O]/[P] = 3.08 for the Ln^3+^-containing ones. However, as will be considered in the light of the XPS results for the O 1s region below, the Ln^3+^-containing glasses exhibited distinct proportions for the oxygen atoms in different environments. As a key aspect of interest, the radius for the different cations decreases continuously as Ba^2+^ > Nd^3+^ > Gd^3+^ > Yb^3+^, which impacts the ionic field strengths considerably as Yb^3+^ > Gd^3+^ > Nd^3+^ ≫ Ba^2+^. This latter property trend of the Ln^3+^ ions will become the subject of attention for interpreting thermomechanical differences (vide infra).

### XPS

**Figure 4:**
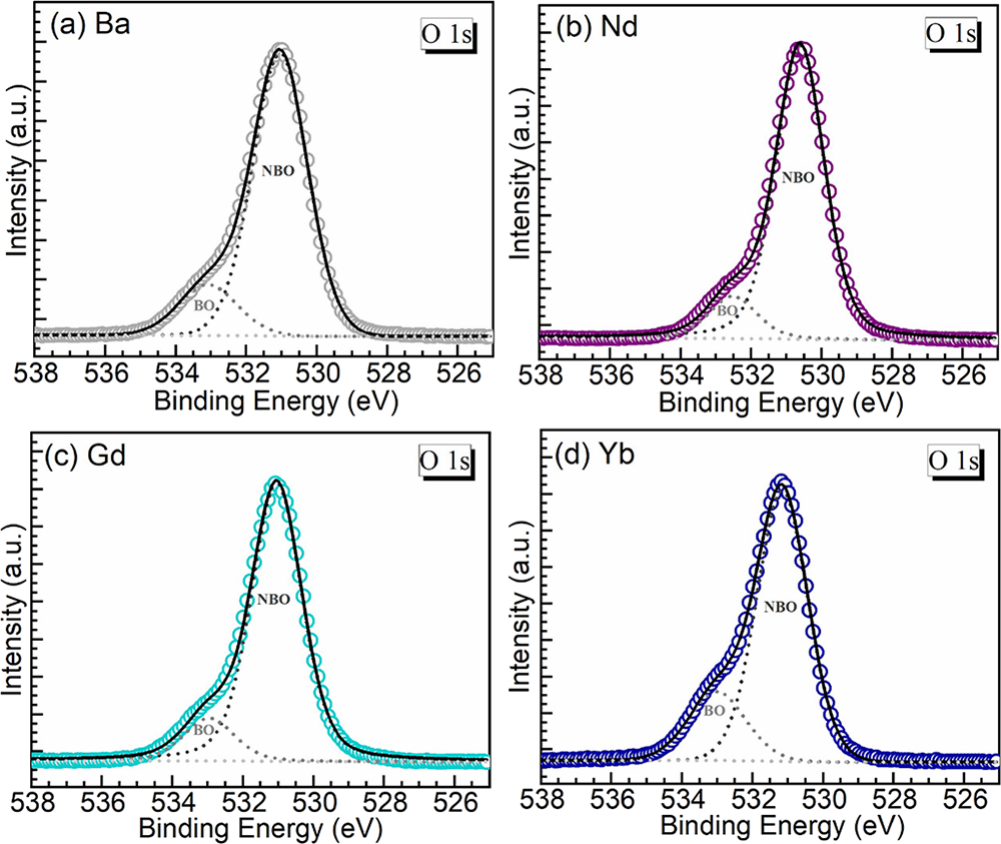
(a–d) XPS O 1s peaks registered for the different glasses studied (open symbols), with the corresponding deconvolutions (results summarized in Table 3) incorporated into the different oxygen species (BO and NBO, dotted curves). The cumulative fits are the solid traces.

To start the assessment of structural properties, we first examine the oxygen bonding environment in the glasses by XPS. This facilitates evaluating the impact of Ln^3+^ ions on the PO_4_ tetrahedra chains, which can be useful in the interpretation of Raman spectroscopy results. Even though XPS is a surface analysis technique, following proper cleaning procedures, the surface of glass materials can be considered representative of the bulk.^27^ Specifically, O 1s XPS data allows for the distinction between bridging oxygens (BOs) in the glass network and nonbridging oxygens (NBOs) interacting with network modifiers; the different proportions of these can be then linked to the degree of polymerization.^27,28^ The O 1s XPS data obtained for the different glasses following the Ar^+^ sputtering for cleaning the surfaces are shown in Figure 4a–d. The experimental spectra displayed peaks at about 531.1, 530.6, 531.1, and 531.2 eV for the Ba, Nd, Gd, and Yb glasses, respectively. In addition, the spectra all exhibit a shoulder toward the high binding energy (BE) side. This latter feature corresponds to BOs (P–O–P) in the structural network, while the dominant peak at lower BE reflects the presence of terminal NBOs (P–O^–^) interacting with metal cations.^27−29^ Hence, the spectra were deconvoluted into the two oxygen contributions, and the resulting bands are also presented overlaid with the experimental traces and the cumulative fits in each panel in Figure 4. The corresponding parameters of BE, full width at half-maximum (fwhm), and % relative area are then summarized in Table 3. As a key parameter deduced from the areas of the two bands, the relative amounts of NBOs were estimated at 83.6, 87.3, 86.6, and 77.8%, for the Ba, Nd, Gd, and Yb glasses, respectively. The NBO content clearly increases with the replacement of 4 mol % BaO with Nd_2_O_3_ and Gd_2_O_3_, thus implying that these induced depolymerization. However, the result is similar for the Nd and Gd glasses, which rounds to ∼87% for both. On the other hand, the Yb glass shows in Table 3 a relative amount of NBOs of 77.8%, which is even lower than the Ba host with 83.6%. It is worth mentioning at this juncture that the content of dopants being equal at 4 mol % in the Ln_2_O_3_-containing glasses, the three of them would contribute twice the amount of Ln^3+^ ions as an equal number of moles of BaO. In addition, the [O]/[P] ratios for the Nd, Gd, and Yb glasses being 3.08 (Table 2), one would expect that because of this, all else being equal, the 4 mol % of Nd_2_O_3_, Gd_2_O_3_ and Yb_2_O_3_ would have a similar effect on the BO and NBO contents in the glass structure. However, we notice that the key aspects of the ionic radius and field strength of the trivalent lanthanides in Table 2 differ considerably. Therefore, while the larger radii Nd^3+^ and Gd^3+^ ions (Table 2) promoted in a similar manner the occurrence of a larger number of shorter phosphate chains in the Nd and Gd glasses compared to the Ba host, the smaller Yb^3+^ ions with high field strength did not depolymerize the glass but rather promoted the opposite. The Yb glass in Table 2 having a lower density than the Gd glass may be connected to this.

**Table 3:** O 1s BE, FWHM, and % Relative Area for the Different Oxygen (BO—Bridging Oxygen; NBO—Nonbridging Oxygen) Components in the Various Glasses, as Estimated from Decomposing the XPS Spectra (Figure 4)

glass	component	O 1s XPS		
		BE (eV)	fwhm (eV)	% area
Ba	BO	533.1	2.0	16.4
	NBO	531.1	1.8	83.6
Nd	BO	532.5	1.6	12.7
	NBO	530.6	1.6	87.3
Gd	BO	533.0	1.7	13.4
	NBO	531.1	1.7	86.6
Yb	BO	533.0	1.9	22.2
	NBO	531.2	1.7	77.8

**Figure 5:**
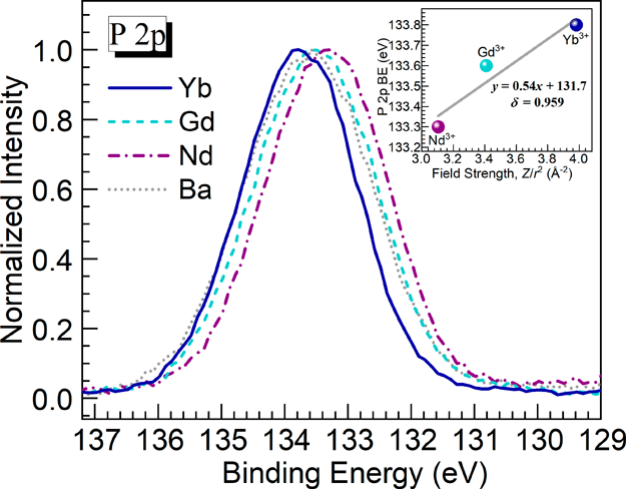
Normalized XPS P 2p peaks for the different glasses under study. The inset is a plot of the P 2p peak BE (Table 4) as a function of the ionic field strength of the Ln^3+^ ions (Table 2) for the Nd, Gd, and Yb glasses; the solid line is a linear fit to the data (equation and correlation coefficient, δ, displayed).

Continuing with the XPS evaluation, we turn our attention to the P 2p peaks obtained for the glasses, as shown in Figure 5. The parameters of the BE and fwhm values deduced are presented in Table 4. It is noticed that the BE for the P 2p peak of the Nd glass (133.3 eV) shifted to a lower value relative to that of the Ba host (133.6 eV). However, it then increased for the Gd (133.6 eV) and Yb (133.8 eV) glasses compared to the Nd glass (133.3 eV). An overall trend of decreasing band widths was also reflected for the Ln^3+^-containing glasses relative to the Ba reference glass as seen in Table 4. Gresch et al.^30^ performed XPS characterization of sodium phosphate glasses also paying attention to the BE shifts of the P 2p and P 2s. The authors observed that the BE of the peaks decreased as the content of the network modifier (Na_2_O) increased, thus connecting the behavior with a larger number of NBO (P–O^–^) increasing electron density toward phosphorus.^30^ The present result for the BE of the Nd glass (133.3 eV) being shifted to lower energy compared to the Ba glass (133.6 eV) seems to agree with this interpretation since the Nd glass also showed a higher NBO content (Table 3). However, the BE of the P 2p peak for the Gd glass of 133.6 eV is the same as the Ba host, while the Gd glass has higher NBO content. Thus, the data suggests that another factor is affecting the BE shift. The authors then hypothesized that the increasing field strength of the Ln^3+^ cations may also impact the BE shift. To examine this further, the P 2p peak positions (Table 4) were plotted as a function of the ionic field strength (Table 2), as shown in the inset of Figure 5. The regression analysis performed on the data yielded a correlation coefficient, δ, of 0.959. Even though striking linearity was not obtained, it is still supported that the P 2p BE shifts to higher values with increasing field strength of the Ln^3+^ ions. This can be explained in terms of the Ln^3+^ cations pulling electron density from the NBOs. This in turn would decrease electron density around phosphorus, and consequently, the BE of the 2p electrons increases. This would harmonize with the early study from Pelavin et al.^31^ on the BE of P 2p electrons in various phosphorus-containing compounds where correlations were sought with the charge of the phosphorus atoms. Overall, the XPS data indicate that the structural effects are not merely driven by the overall [O]/[P] ratios, which are the same for the Ln^3+^-containing glasses (Table 2), but rather the distinct environment of oxygen atoms impacting phosphorus in the structural network. Having thus set the background with respect to basic physical properties and insights from O 1s and P 2p XPS, the Raman spectroscopy results are considered next.

**Table 4:** P 2p Peaks BE and FWHM Values Obtained for the Different Glasses from XPS Spectra (Figure 5)

glass	P 2p XPS	
	BE (eV)	fwhm (eV)
Ba	133.6	2.4
Nd	133.3	2.3
Gd	133.6	2.3
Yb	133.8	2.2

### Raman Spectroscopy

**Figure 6:**
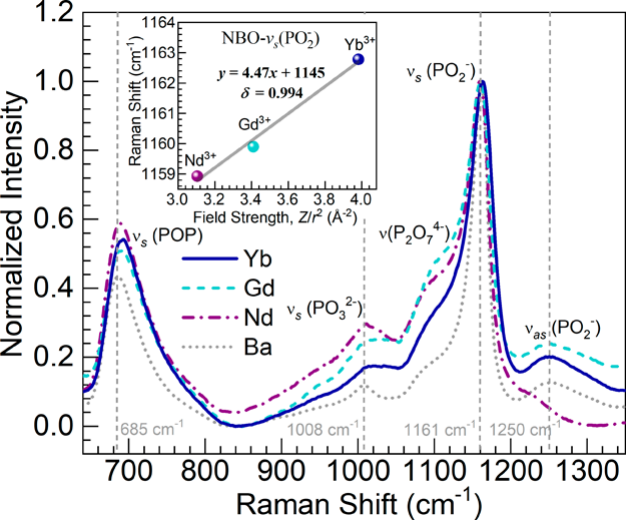
Normalized Raman spectra for the various glasses within the 640–1350 cm^–1^ spectral range for comparison; main spectroscopic features in the Ba glass as the reference are indicated (vertical dashed lines–wavenumbers displayed). The inset is a plot of the position of the NBO-ν_*s*_(PO_2_^–^) Raman band (Table 5) as a function of the ionic field strength of the Ln^3+^ ions (Table 2) for the Nd, Gd, and Yb glasses; the solid line is a linear fit to the data (equation and correlation coefficient, δ, displayed).

The Raman spectra obtained for the different glasses are shown in Figure 6. Following baseline subtraction, the spectra were normalized with respect to the strongest band within the 640–1350 cm^–1^ spectral range considered. The assignment for the various features is achieved according to the literature as reported for similar glasses.^15,22,23,32^ Taken as the reference, the binary 50P_2_O_5_–50BaO host glass exhibits a band around 685 cm^–1^ toward the low energy region, ascribed to the in-chain symmetric stretching vibrations in P–O–P bridges, ν_s_(POP), in *Q*^2^ tetrahedral units (PO_4_ tetrahedra with 2 BOs). The small feature observed around 1008 cm^–1^ is credited to the symmetric stretch, ν_s_(PO_3_^2–^), in NBOs pertaining to *Q*^1^ units (PO_4_ tetrahedra with 1 BO). Then the Ba glass shows the most intense band around 1161 cm^–1^ in connection with the out-of-chain symmetric stretching in PO_2_^–^ groups, ν_s_(PO_2_^–^), that take place in NBOs belonging to the *Q*^2^ units. Lastly, the asymmetric stretching vibrations in these, ν_as_(PO_2_^–^), are expressed at around 1250 cm^–1^ in the Ba host.

It is observed for the Nd and Gd glasses in Figure 6 that a feature is expressed toward the lower frequency side of the ν_s_(PO_2_^–^) band around 1100 cm^–1^, suggesting the manifestation of stretching vibrations of *Q*^1^ units in P_2_O_7_^4–^ dimers.^33^ An increased intensity of the ν_s_(PO_3_^2–^) band is also noticeable for these two glasses. These considerations point to Nd^3+^ and Gd^3+^ ions inducing glass depolymerization relative to the Ba host glass, which is also supported by the O 1s XPS results (vide supra). The Yb glass, on the other hand, does not show the enhancement of ν_s_(PO_3_^2–^) and ν(P_2_O_7_^4–^) features in the same way. This appears to be consistent with the lower content of NBOs in the Yb glass from XPS relative to the Nd and Gd glasses (Table 3). However, the Yb glass still shows the features augmented relative to the Ba host glass with higher NBO content, so the effect cannot be merely explained by the oxygen speciation results. The ν_as_(PO_2_^–^) feature still appears prominently around the Gd and Yb glasses in the vicinity of 1250 cm^–1^. Yet, for the Nd glass, it appears to be weakened and shifted to lower frequencies toward the ν_s_(PO_2_^–^) band. A similar outcome has been observed in Raman spectra obtained for other Nd-doped phosphate-based glasses, interpreted as an effect from Nd^3+^ ions decreasing bond covalency and impacting the PO_2_ bond asymmetric stretch considerably.^34,35^ Furthermore, toward the low-frequency region, the ν_s_(POP) band (BO-related) shows some differences among the glasses, for instance, concerning position and intensity relative to the ν_s_(PO_2_^–^) band (NBO-related). To aid in the evaluation, presented in Table 5 are the peak positions of the ν_s_(POP) and ν_s_(PO_2_^–^) bands as parameters of interest. The former BO-related band is observed to exhibit a trend toward higher frequencies while exhibiting some broadening for the Nd, Gd, and Yb glasses compared to the Ba reference. This type of behavior has been associated with depolymerization effects in phosphate glasses.^15,22,32^ Shorter PO_4_ tetrahedra chains yield higher frequency components of the ν_s_(POP) band, while the broadening points to higher heterogeneity.^15,32^ This interpretation could be applied to the Nd and Gd glasses, given that XPS results indicated a higher content of NBO in these glasses (Table 3). Yet, the explanation does not suffice for the Yb glass with the lowest NBO content. The high-field strength of Yb^3+^ cations thus appears to be impacting the shift of the ν_s_(POP) band to higher frequencies. These in-chain vibrations are impacted by the cationic environment over long-chain segments.^36^ The effect observed may then be linked to the smaller ionic size of the Yb^3+^ ions, resulting in smaller P–O–P bond angles causing the shift and broadening given that depolymerization was not supported by XPS.

**Table 5:** Spectral Positions for the BO-ν_s_(POP) and NBO-ν_s_(PO_2_^–^) Raman Bands (Figure 6) for Different Glasses

glass	BO-ν_s_(POP) (cm^–1^)	NBO-ν_s_(PO_2_^–^) (cm^–1^)
Ba	685	1161
Nd	688	1159
Gd	693	1160
Yb	693	1163

Furthermore, the ν_s_(PO_2_^–^) band which relates to NBOs shifts toward lower wavenumber for the Nd glass relative to that for the Ba glass but thereafter shifts to higher values for the Gd and Yb glasses (Table 5). The ν_s_(PO_2_^–^) band is known to be sensitive to the degree of covalency and the interaction with the cation modifiers in the glass network.^32,37^ We consider in this context that the NBO–Ba^2+^ bonds with high ionicity become replaced by NBO–Nd^3+^ bonds with more covalent character given the considerably higher electronegativity of neodymium. In turn, the higher covalency of the oxygen–neodymium bond would lead to more ionicity of the phosphorus–oxygen bond, which could explain the red shift of the band in the Nd glass relative to that in the Ba host. On the other hand, a shift to higher energies of the ν_s_(PO_2_^–^) band with the decrease in radius of the Ln^3+^ ion is consistent with the report from Sendova et al.,^16^ indicating stronger *Q*^2^ NBO bonding also linked to the glass transition activation energy. Interestingly, in a scenario analogous to the current results, it was noticed by Koo et al.^37^ for binary copper phosphate glasses that the interpretation of depolymerization pointed by Raman spectroscopy was not consistent with XPS. Thus, rather than a mere change in phosphate chain length, the authors considered local bonding to be influenced by the different ionic size of Cu^+^ relative to Cu^2+^ and the resulting field strength.^37^ Hence, we consider the impact of ionic field strength of the trivalent lanthanides on the ν_s_(PO_2_^–^) band, which is sensitive to the cation modifiers.^16,37^ To explore this further, the ν_s_(PO_2_^–^) band position (Table 5) was plotted as a function of Ln^3+^ ionic field strength (Table 2) for the Nd, Gd, and Yb glasses, as shown in the inset of Figure 6. A linear correlation is indicated by the regression analysis performed on the data, where the correlation coefficient, δ, was 0.994. It supports the finding that the shift to higher frequencies of the ν_s_(PO_2_^–^) band follows the increasing field strength of the Ln^3+^ cations. In this connection, it is worth pointing out that the P 2p XPS data (Figure 5, Table 4) signaled that the increase in cation field strength decreased electron density around phosphorus, thus increasing the peak BE. Accordingly, it may be suggested that this electron withdrawal effect favors the strengthening of the out-of-chain P–O bonds in *Q*^2^ units (e.g., increasing the covalent character). Following the assessment of structural properties performed, the thermal behavior studied by dilatometry is considered next.

### Dilatometry

**Figure 7:**
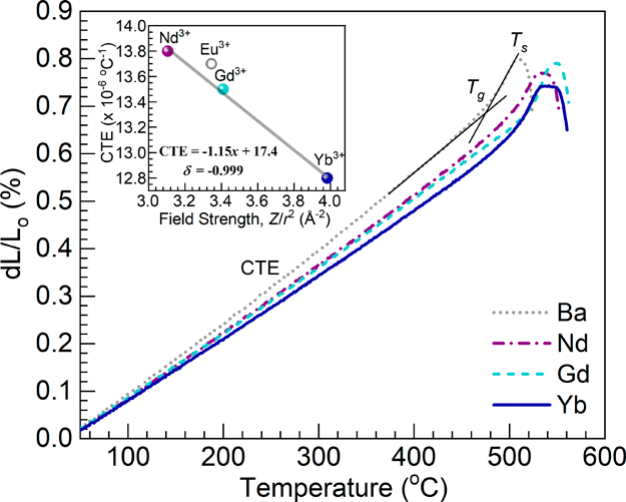
Dilatometric profiles were obtained for the different glasses. The inset is a plot of the CTE values estimated (Table 6) as a function of the ionic field strength of the Ln^3+^ ions (Table 2) for the Nd, Gd, and Yb glasses; the solid line is a linear fit to the data (equation and correlation coefficient, δ, displayed). The open circle in the inset is the data point obtained for Eu^3+^ based on the calculated field strength and the CTE reported in ref (22).

Figure 7 shows the dilatometric profiles obtained for the different glasses under consideration. Starting with the Ba glass as the reference, the evolution of the linear expansion profiles d*L*/*L*_o_ (%) vs temperature was used for the extraction of the parameters of coefficient of thermal expansion (CTE), glass transition temperature (*T*_g_), and softening temperature (*T*_s_).^22,38^ The linear CTE values were determined in the 50–400 °C range consistently, whereas the *T*_g_ was estimated from the intersecting lines approach (illustrated with the Ba glass in Figure 7) and *T*_s_ from the peak temperature within the expansion region.^38^ The different values obtained are summarized in Table 6 (the uncertainties in the various parameters are assumed as reported in ref (38) ). The *T*_g_ and *T*_s_ values first increase for the Nd and Gd glasses relative to that for the Ba host but then decrease for the Yb relative to that for the Gd glass. This behavior resembles the density results (Table 2) as well as XPS oxygen speciation (Table 3) wherein the Yb glass deviated from the other lanthanide-containing glasses. The decrease in *T*_g_ and *T*_s_ values for the Yb glass relative to that for Gd may then be related to the lower NBO content. The specific concentration dependence of the physicochemical properties of the different Ln^3+^-containing glasses will be the subject of separate studies to encompass a wide range of experimental techniques including differential scanning calorimetry measurements. Nevertheless, the current data suggest that the Yb^3+^ ions partake in a unique manner within the phosphate glass structure.

**Table 6:** Values of Linear Coefficient of Thermal Expansion (CTE, Estimated in the 50–400 °C Range), Glass Transition Temperature (*T*_g_), and Softening Temperature (*T*_s_) Estimated from Dilatometry Data (Figure 7)

glass	CTE ± 0.1 (× 10^–6^ °C^–^^1^)	*T*_g_ ± 4 (°C)	*T*_s_ ± 3 (°C)
Ba	15.0	475	508
Nd	13.8	494	531
Gd	13.5	521	545
Yb	12.8	498	537

As a focal point of the present work, the CTE values obtained (Table 6) clearly decrease for the glasses in the following order: Ba > Nd > Gd > Yb. The CTE for the Ba reference glass of 15.0 × 10^–6^ °C^–^^1^ is in reasonable agreement with the reported value of 14.9 × 10^–6^ °C^–^^1^ for an equivalent glass prepared using a high-purity alumina crucible.^22^ Furthermore, it is observed in Table 6 that replacing the 4 mol % of BaO by Nd_2_O_3_ already produces a considerable decrease in the CTE. The CTE of the Nd glass of 13.8 × 10^–6^ °C^–^^1^ can be considered on the high side compared to the values reported for different Nd phosphate glasses having multiple components some of which tend to decrease the CTE.^19,39^ It is nonetheless close to the reported within the 20–300 °C range for Schott’s LG-770 high-energy/high-power (HEHP) laser glass^18^ of 13.4 × 10^–6^ °C^–^^1^, which is of the aluminophosphate type. Table 6 also shows that the effect of CTE reduction is progressive for equal amounts of Gd_2_O_3_ and Yb_2_O_3_ compared to Nd_2_O_3_. With respect to gadolinium, Xu et al.^40^ reported a trend of decreasing CTE (within the 9.08 × 10^–6^–8.76 × 10^–6^ °C^–^^1^ range) for the glass system with 60P_2_O_5_–25Bi_2_O_3_–(10 – *x*)CaO–5Sb_2_O_3_–*x*Gd_2_O_3_ (*x* = 0, 1, 2, 3, 4 mol %) composition, which was interpreted in terms of the high ionic field strength of Gd^3+^. Li et al.^41^ studied phosphate glasses of 12CaO–20Fe_2_O_3_–68P_2_O_5_ with 10 mol % Ln_2_O_3_ (Ln = Y, La, Nd, Sm, and Gd) compositions and noticed that the CTE was lower for Ln = Y, Sm, and Gd also discussed based on high field strengths. Further, in their study of doped Si–Mg–O–N glasses, Lofaj et al.^15^ found that the CTE of the glasses decreased approximately linearly for the various lanthanides studied, which included neodymium, gadolinium, and ytterbium, with the field strength of the cations. Consistent with such report, the small radius Yb^3+^ ions herein appear particularly suited for achieving the low CTE. Remarkably, low CTE values have been also reported for Yb-doped aluminophosphate glasses likely benefiting from the high field strength of Yb^3+^ ions, besides other constituents such as Al^3+^.^42,43^

Considering the O 1s XPS data discussed herein, the degree of depolymerization does not seem to be the main factor impacting the CTE. This is because the Nd and Gd glasses had higher NBO content than the Ba glass (Table 3) and yet exhibited lower CTE values than the Ba reference, whereas the Yb glass then had lower NBO content but showed the lowest CTE. This contrasts with the changes observed for bismuth borate glasses of (25 + *x*)Bi_2_O_3_–15BaO–10Li_2_O–(50 – *x*)B_2_O_3_ (*x* = 0, 10, 20, 30 mol %) compositions where the CTE values increased, while the content of NBO assessed by XPS also increased.^38^ Similarly, in their work on 10CaF_2_–(29.5 – 0.4*x*)CaO–(60–0.6*x*)B_2_O_3_–*x*TeO_2_–0.5Yb_2_O_3_ (*x* = 10, 16, 22, 31, 54 mol %) glasses, de Oliveira Lima^44^ also noticed that the CTE increased with the amount of TeO_2_ discussed in terms of the presence of NBOs decreasing glass connectivity. The increase of NBO concentration leading to higher CTE values was also pointed out by Li et al.^39^ on their report of Nd-doped phosphate glass. Interestingly, a shift toward higher frequencies of the main Raman bands was also observed for the Nd-doped glass with lower CTE (NAP2 glass) relative to the one with higher CTE (N31 glass).^39^ Further, the CTE data are herein consistent with the Raman spectroscopy results analysis assisted by XPS supporting a strong interaction of the Ln^3+^ ions with *Q*^2^ NBO bonds (Figure 6, inset). Thus, considering the concentration of the lanthanide oxides is equivalent in the Ln^3+^-containing glasses and in agreement with the structural characterizations, we turn our attention to the variable of ionic radius and the consequent field strength.^15,17,39−41^ Therefore, the CTE values (Table 6) were plotted as a function of the Ln^3+^ ionic field strength (Table 2) for the Nd, Gd, and Yb glasses as shown in the inset of Figure 7. A linear correlation is evident by the regression analysis performed, which yielded a correlation coefficient δ = −0.999. We also consider at this point the CTE value of 13.7 × 10^–6^ °C^–1^ reported for the glass with 50P_2_O_5_–46BaO–4Eu_2_O_3_ nominal composition melted in a corundum crucible.^22^ For Eu^3+^, the ionic radius in 6-fold coordination is 0.947 Å,^14^ resulting in a corresponding field strength of 3.345 Å^–2^ calculated by eq 1. This data point is added in the inset of Figure 7 (open circle) for comparison, which is seen to lie between Nd^3+^ and Gd^3+^. Interestingly, the *T*_g_ and *T*_s_ values estimated from dilatometry for the 50P_2_O_5_–46BaO–4Eu_2_O_3_ glass in ref (22) were 514 and 538 °C, respectively, which are also between the obtained herein for the Nd and Gd glasses (Table 6). It is overall then supported that the decrease in CTE takes place following the increasing field strength of the Ln^3+^ cations. Besides concurring with other works on lanthanide field strength effects,^15,20,39−41^ the present results are also consistent with the report by Hayden et al.^17^ showing a significant correlation for the CTE with the average field strength of the alkali and alkaline earth cations in Nd laser glass. It then appears that the high field strengths of the trivalent lanthanides promote the increased rigidity of the glasses, leading to tighter networks with lesser susceptibility to expansion with temperature.

## Conclusions

In brief, the melting technique was employed to synthesize phosphate glasses containing Nd^3+^, Gd^3+^, and Yb^3+^ ions as technologically relevant lanthanides to pursue a composition–structure–property study focusing ultimately on the dilatometric behavior. The glasses were prepared with 50P_2_O_5_–46BaO–4Ln_2_O_3_ nominal compositions (mol %) with Ln = Nd, Gd, and Yb for the comparative assessment, with the 50P_2_O_5_–50BaO binary made as a reference. The characterizations performed by XRD and optical spectroscopy measurements confirmed the noncrystalline nature of the glasses and the occurrence of the different Ln^3+^ ions by their corresponding absorption/emission features. Density and physical properties were then evaluated, where the densities and molar volumes of the Ln^3+^-containing glasses were higher than those of the barium phosphate host. A slight decrement in density was observed for Yb^3+^ relative to Gd^3+^; however, the Ln^3+^–Ln^3+^ mean distances were consistently determined for the glasses around 9.5 Å. Oxygen speciation analysis carried out by XPS yielded an increase in the NBO content for the larger radii Nd^3+^ and Gd^3+^ ions relative to the undoped host but a decrease for Yb^3+^. Moreover, phosphorus XPS analysis supported the hypothesis that the P 2p binding energies of the glasses increased with the cation field strength of the lanthanides. Raman spectroscopy further employed to interrogate structural properties showed that the effect of Nd^3+^ and Gd^3+^ ions generally agreed with a depolymerization effect, whereas Yb^3+^ was considered to influence the Raman features in connection with its high field strength. However, the NBO-ν_*s*_(PO_2_^–^) band position shifting to higher frequencies was observed to correlate linearly with the field strengths of the Ln^3+^ ions, supporting a bond strengthening effect with smaller ionic radii. The thermal behavior evaluated by dilatometry ultimately revealed a steady decrease in the thermal expansion coefficient with the decreasing radius of the cationic modifiers. Remarkably, a linear correlation was observed for the decrease in the thermal expansion coefficient of the glasses with the ionic field strength of the Nd^3+^, Gd^3+^, and Yb^3+^ ions. Comparison with reported dilatometry data for a similar glass with Eu^3+^ was found to be in line with the interpretation, with Eu^3+^ found to lie between Nd^3+^ and Gd^3+^ concerning thermal parameters as well as ionic radius and field strength. It is overall supported that the increased field strength of the trivalent lanthanides increases the rigidity of the glasses making them less susceptible to thermal expansion likely through the influence of the cationic modifiers interacting with the oxygen terminals in the phosphate structure.
